# Src homology 2 (SH2) domain containing protein tyrosine phosphatase-1 (SHP-1) dephosphorylates VEGF Receptor-2 and attenuates endothelial DNA synthesis, but not migration*

**DOI:** 10.1186/1750-2187-3-8

**Published:** 2008-03-31

**Authors:** Resham Bhattacharya, Junhye Kwon, Enfeng Wang, Priyabrata Mukherjee, Debabrata Mukhopadhyay

**Affiliations:** 1 Department of Biochemistry and Molecular Biology and Mayo Clinic Cancer Center, College of Medicine, Mayo Clinic, Rochester, MN 55905, USA

## Abstract

**Background:**

Vascular endothelial growth factor receptor-2 (VEGFR-2, KDR), a receptor tyrosine kinase, regulates mitogenic, chemotactic, hyperpermeability, and survival signals in vascular endothelial cells in response to its ligand vascular permeability factor/ vascular endothelial growth factor (VPF/VEGF). SHP-1 is a protein tyrosine phosphatase known to negatively regulate signaling from receptors such as EGF receptor, IL3 receptor, erythropoietin receptor and also KDR. However, the mechanism by which SHP-1 executes KDR dephosphorylation, the targeted tyrosine residue(s) of KDR and also overall downstream signaling or phenotypic change(s) caused, is not defined.

**Results:**

Here, we have demonstrated that KDR and SHP-1 are constitutively associated and upon VEGF treatment, the phosphatase activity of SHP-1 is stimulated in a c-Src kinase dependent manner. Knockdown of SHP-1 by siRNA or inhibition of c-Src by an inhibitor, results in augmented DNA synthesis perhaps due to increased phosphorylation of at least three tyrosine residues of KDR 996, 1059 and 1175. On the other hand, neither tyrosine residue 951 of KDR nor VEGF-mediated migration is affected by modulation of SHP-1 function.

**Conclusion:**

Taken together our results define the tyrosine residues of KDR that are regulated by SHP-1 and also elucidates a novel feed back loop where SHP-1 is activated upon VEGF treatment through c-Src and controls KDR induced DNA synthesis, eventually leading to controlled angiogenesis.

## Background

Angiogenesis, the sprouting of new blood vessels from pre-existing endothelium is a fundamental feature of both normal physiology and pathologic states including coronary heart disease, diabetes, retinopathy and cancer [1-4]. The growth factor VEGF-A is a key regulator of physiologic and pathologic angiogenesis [5]. VEGF was identified due to its ability to induce vascular hyperpermeability but has since been recognized as a potent inducer of endothelial proliferation, migration and survival. VEGF also acts as a proinflammatory cytokine and induces the expression of a number of molecules implicated in regulating angiogenesis [6,7].

The effects of VEGF and its family of proteins are mediated by three structurally related receptor tyrosine kinases namely VEGFR1/Flt-1, VEGFR-2/Flk-1/KDR, VEGFR3/Flt-4 [8-12]. Among these, KDR has emerged as the main receptor mediating VEGF effects such as endothelial cell proliferation, migration and proinflammatory activation. In contrast, Flt-1 is thought to mediate inhibitory and/or decoy effects in endothelial cells [13,14]. Flt-4 is mainly expressed in lymphatics and regulates lymphangiogenesis [12]. The importance of VEGF/KDR axis is accentuated by the fact that increased levels of both ligand and receptor are found in tumor cells as well as stroma [15-19].

Src homology 2 (SH2) domain-containing protein tyrosine phosphatase (SHP) -1 and -2 are non-receptor protein tyrosine phosphatases (PTPs). Expression of SHP-1 is restricted to hematopoietic cells whereas SHP-2 is more widely expressed [20]. SHP-1 has been proposed to be a candidate tumor suppressor gene in lymphoma, leukaemia and other cancers [21]. Evidence for the differing roles of SHP-1 and SHP-2 in cell signaling has come from the study of mice lacking functional SHP-1 or SHP-2. The SHP-1 gene mutated motheaten (me) mice display severe haematopoietic disruption with chronic inflammation and systemic autoimmunity and die from hemorrhagic pneumonitis [22,23]. Thus the results provide strong evidence for a major role of this phosphatase in the negative regulation of cell function. Targeted disruption of the SHP-2 gene results in embryonic lethality of homozygous mutant mice however generation of chimeric mice from homozygous SHP-2 mutant ES cells and wild-type embryos determined a role for SHP-2 in blood cell development [24,25]. Other studies have demonstrated a role for SHP-2 in positively regulating signaling downstream of the insulin receptor, platelet derived growth factor receptor (PDGFR) and fibroblast growth factor receptor (FGFR) (reviewed in [26]. The SHP-1 enzyme contains two tandem SH2 domains at the N-terminus, followed by the catalytic domain and a C-terminal tail. The C-terminal tail contains multiple sites of tyrosine and serine phosphorylation and this part of the protein has been proposed to have an important regulatory function [20].

A number of studies have shown that SHP-1 negatively regulates signaling of receptors such as the EGF receptor, IL3 receptor, erythropoietin receptor and KDR [27-29]. Studies have also suggested that SHP-1 is a negative regulator of angiogenesis *in vivo *because SHP-1 deficient mice are resistant to TIMP-2 inhibition [30]. SHP-1 has also been shown to co-precipitate with KDR upon stimulation with VEGF and overexpression of SHP-1 resulted in impairment of VEGF mediated KDR autophosphorylation and ERK activation [29]. However, the signaling mechanism by which this inhibition takes place remains to be elucidated. Here, we have clearly defined the respective tyrosine residues of KDR that are regulated by SHP-1 and hence affect downstream signaling. We have also demonstrated that KDR and SHP-1 are constitutively associated and upon VEGF treatment the phosphatase activity of SHP-1 is stimulated in a c-Src kinase dependent manner. Tyrosine phosphorylation and activation of SHP-1 is regulated by c-Src. Knockdown of SHP-1 by siRNA or inhibition of c-Src results in amplified proliferation that may be due to increased phosphorylation of at least three tyrosine residues on KDR such as 996, 1059 [31] and 1175 [32]. Interestingly, VEGF-mediated migration is not affected as well as tyrosine residue 951 on KDR is unchanged [31]. Overall, our results define the residues on KDR that are regulated by SHP-1 and also elucidate a novel feed back loop by which SHP-1 is activated upon VEGF treatment through c-Src kinase and attenuates KDR mediated DNA synthesis.

## Results

### SHP-1 Co-Precipitates with KDR and Src

**Figure 1:**
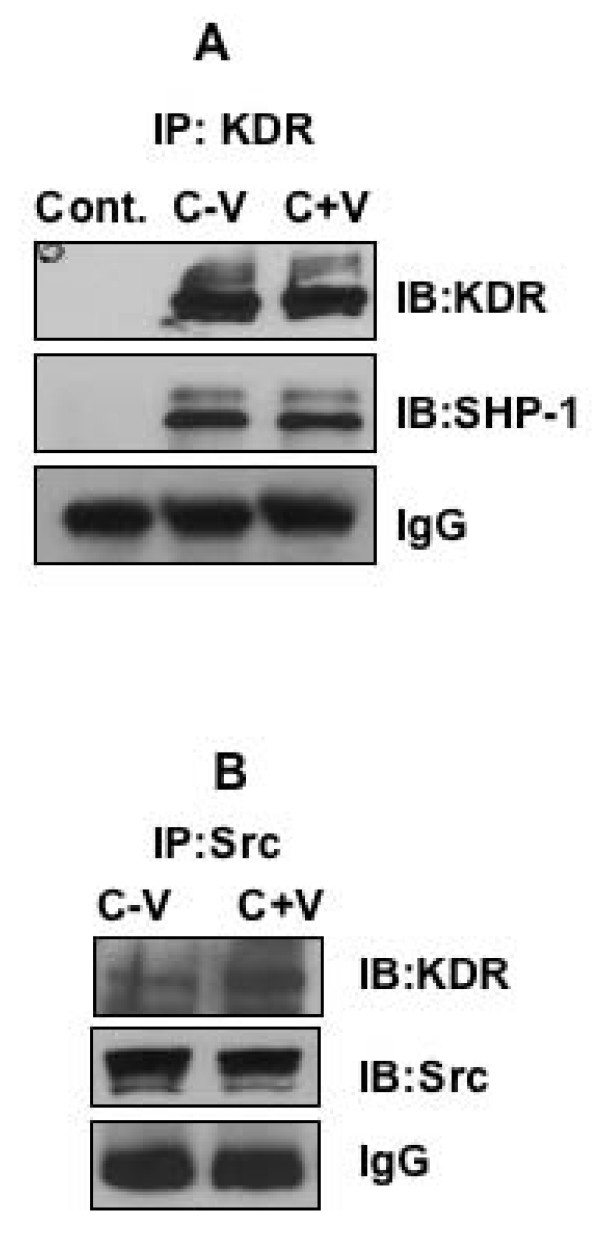
**KDR, c-Src and SHP-1 co-precipitate and are part of one complex**. (A) Serum starved HUVEC was treated with or without VEGF 10 ng/ml for 5 min and immunoprecipitated with antibody against KDR and immunoblotted with SHP-1 antibody. The first lane represents negative control (rabbit IgG). (B) Serum starved HUVEC was treated with or without VEGF 10 ng/ml for 5 min and immunoprecipitated with antibody against c-Src and immunoblotted with KDR and Src antibody.

Initially, co-precipitation studies were carried out to determine the role of SHP-1 in KDR mediated signaling. As shown in Fig 1A, we observed that KDR co-immunoprecipitated with SHP-1 in HUVEC with or without VEGF treatment. The specificity of the SHP-1 antibody was determined using the catch and release column (see Additional file 1). Our results are in contrast to two previous reports that suggest the association of SHP-1 with KDR upon induction with VEGF [33,34]. However, we have repeatedly found constitutive association between KDR and SHP-1. We have speculated that the association of SHP-1 with KDR occurs through the SH2 domains of SHP-1 but activation of the phosphatase activity occurs only when VEGF treatment causes availability of phosphorylated residues on KDR. KDR also co-immunoprecipitated with c-Src kinase in HUVEC treated with or without VEGF (Fig 1B). Interestingly, the association of KDR with c-Src significantly increased upon treatment with VEGF. Next, we addressed the question whether VEGF treatment could increase the phosphatase activity of SHP-1 and how c-Src kinase influences this activation?

### Activation of SHP-1 Upon VEGF Stimulation

**Figure 2:**
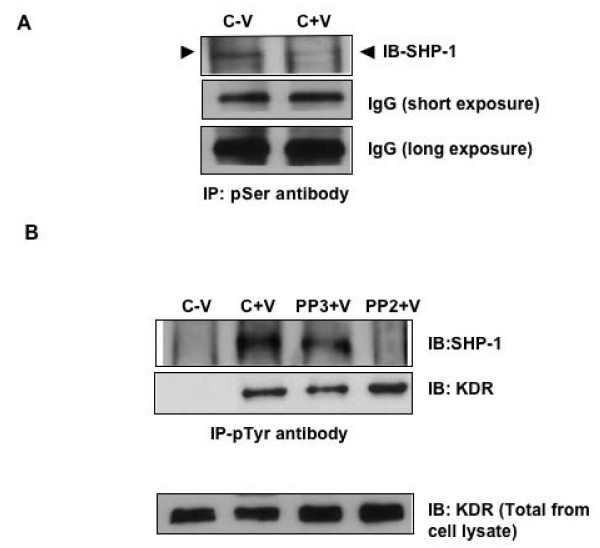
**Serine and tyrosine phosphorylation of SHP-1**. (A) Serum starved HUVEC was treated with or without VEGF 10 ng/ml for 5 min and immunoprecipitated with antibody against phospho-Ser and immunoblotted with HRP conjugated SHP-1 antibody. (B) Serum starved HUVEC was pretreated with PP2 or PP3, 5 uM for 1 h followed by treatment with VEGF 10 ng/ml for 5 min and immunoprecipitated with antibody against phospho-Tyr and immunoblotted with SHP-1 and KDR antibody. Cell lysates from the same sample were also run to show total KDR levels.

It has been previously established that the catalytic activity of SHP-1 is regulated by the phosphorylation of amino acids at the C terminus of the protein [20]. The phosphatase activity of SHP-1 is increased by phosphorylation on Tyr 536 and decreased by phosphorylation on Ser 591. Similarly, we observed that upon stimulation of HUVEC with VEGF, tyrosine phosphorylation of SHP-1 increased whereas serine phosphorylation decreased (Fig 2A,B). The increase in tyrosine phosphorylation and decrease in serine phosphorylation suggested that the phosphatase activity of SHP-1 was activated upon VEGF stimulation. When we utilized PP2, a c-Src kinase inhibitor, VEGF mediated tyrosine phosphoryation of SHP-1 was inhibited (Fig 2B) but not with PP3, the analog of PP2. It should be noted that upon PP2 treatment overall phosphorylation of tyrosine residues on KDR also increased compared to the control. These results suggested that c-Src might play a role in VEGF-mediated SHP-1 phosphatase activation by phosphorylating its tyrosine residue.

**Figure 3:**
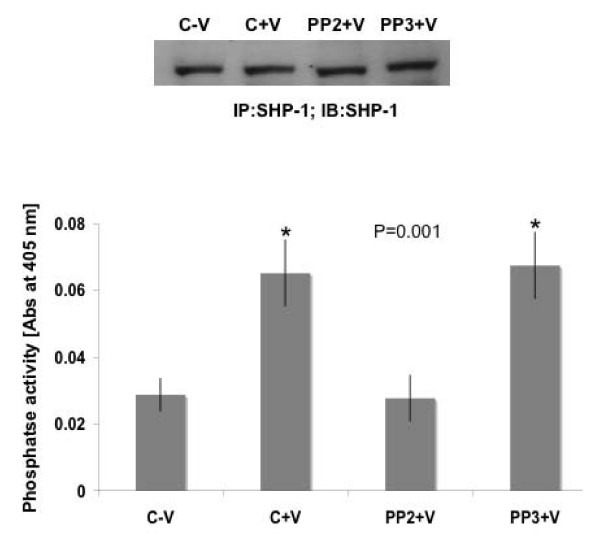
**SHP-1 phosphatase assay**. Phosphatase assay was performed using pNNP. Serum starved HUVEC was pretreated with PP2 or PP3, 5 uM for 1 h followed by treatment with VEGF 10 ng/ml for 5 min and immunoprecipitated with antibody against SHP-1 using, "catch and release column," from Upstate. One part was run on a gel and the other part was used for the phosphatase assay. Data represent average of three independent determinations normalized with respect to negative control (P = 0.001).

Next we examined the status of phosphatase activity of SHP-1 in HUVEC in response to VEGF treatment. As shown in Figure 3, phosphatase activity of SHP-1 (data normalized with respect to negative control) significantly increased after VEGF treatment and decreased after treatment with PP2 but not with PP3. That equal amounts of SHP-1 and not SHP-2 protein immunoprecipitated with the different treatments was also confirmed (Fig 3 and see Additional file 1). We also performed a time course study (0, 5, 15, 30 and 60 min) but did not observe any significant increase in the phosphatase activity of SHP-1 over control beyond 5 min treatment with VEGF (data not shown). However, the question remains why VEGF treatment promotes SHP-1 phosphatase activity? Hence, we postulated that in the absence of SHP-1 we should observe an increase in VEGF mediated signaling in EC.

### SHP-1 is Required for Modulating the VEGF-Mediated DNA Synthesis

**Figure 4:**
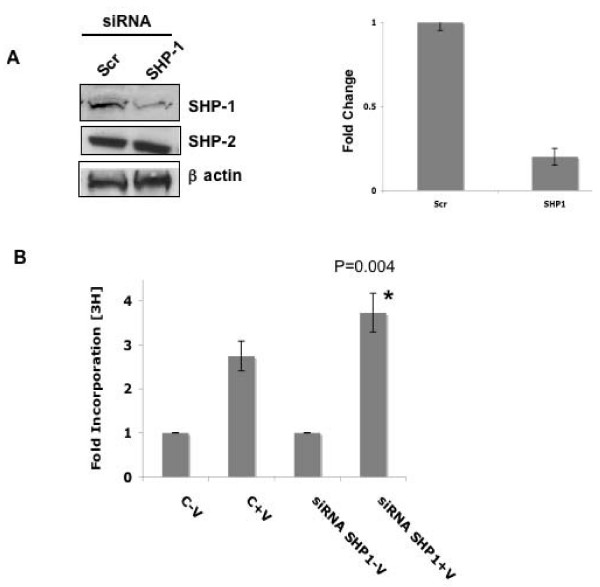
**Knockdown of SHP-1 using siRNA and its effect on DNA synthesis**. (A) HUVEC were transfected with scrambled control or SHP-1 siRNA using oligofectamine and protein levels checked after 48 h. β actin served as a loading control. SHP-2 levels were not affected. NIH Image quantitation data normalized with respect to b actin is shown. (B) HUVEC were transfected with scrambled control or SHP-1 siRNA using oligofectamine. After 48 h, the cells were plated on 24 well plates and [^3^H]thymidine incorporation assay carried out as described in Materials and Methods. Data represent average of five independent determinations each in triplicate (P = 0.004).

Herein, we utilized SHP-1 siRNA to block its expression in HUVEC. The image quantitation data indicate that there was a ~80% decrease in protein levels of SHP-1 after 48 hrs in HUVEC transfected with SHP-1 siRNA as compared to that of scrambled siRNA. The SHP-1 siRNA was specific since there was no change in SHP-2 protein levels (Fig 4A). We further studied VEGF-mediated DNA synthesis in HUVEC in response to 10 ng/ml VEGF by [^3^H]thymidine incorporation after transfection with SHP-1 siRNA. Interestingly, Figure 4B shows that there was a significant and consistent increase in [^3^H]thymidine incorporation by HUVEC when treated with VEGF suggesting further that in the presence of VEGF, SHP-1 functions as an active phosphatase.

### Knockdown of SHP-1 Interferes With VEGF Induced Signaling

**Figure 5:**
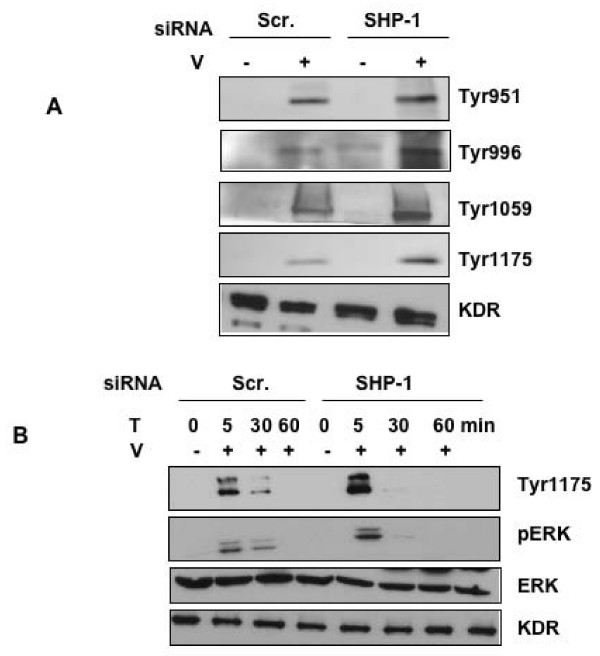
**Effect of SHP-1 knockdown on phosphorylation of KDR and ERK**. (A) HUVEC were transfected with scrambled control or SHP-1 siRNA using oligofectamine. After 48 h the cells were serum starved and treated with or without VEGF 10 ng/ml and immunoblotted with antibodies against p-Tyr of KDR. Total KDR served as a loading control. (B) HUVEC were transfected with scrambled control or SHP-1 siRNA using oligofectamine. After 48 h the cells were serum starved and treated with or without VEGF 10 ng/ml and immunoblotted with antibodies against p-ERK. Total ERK served as a loading control.

**Figure 6:**
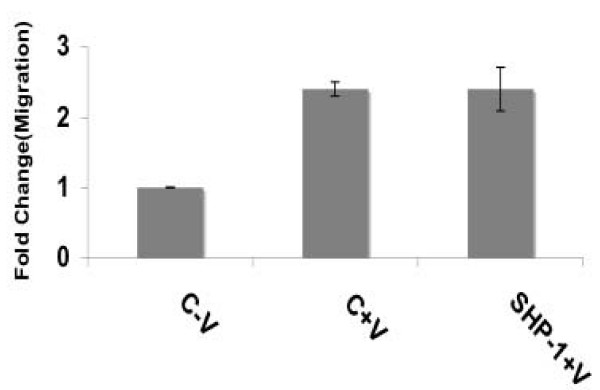
**Effect of SHP-1 knockdown on migration**. HUVEC were transfected with scrambled control or SHP-1 siRNA using oligofectamine. After 48 h, the cells were plated on 6 well plates and wound healing migration carried out as described in Materials and Methods. Data represent average of three independent determinations each in triplicate.

To examine further the signaling components in the proliferation pathways, we examined phospho-KDR as an upstream marker and phospho-ERK as a downstream marker. It has been shown previously that tyrosine residues 996, 1059 and 1175 are phosphoryted in the presence of VEGF and lead to EC proliferation [31,32]. In HUVEC transfected with SHP-1 siRNA, phosphorylation of KDR at Tyr996, 1059 and 1175 increased significantly (Image quantitation data, Additional file 2) as compared to that of scramled siRNA, when treated with VEGF (Fig 5A). However, there is no significant difference in the phosphorylation of KDR at Tyr951 (Fig 5A) (Image quantitation data, see Additional file 2). In addition, no significant difference in migration was observed between scrambled control and SHP-1 siRNA treated HUVEC in the presence of VEGF suggesting that SHP-1 might not influence VEGF-mediated migration (Fig 6 and see Additional file 4). We next performed a time course study, 0–60 min, on siRNA transfected HUVEC treated with VEGF. We observed a significant increase in phosphorylation of ERK and in Tyr1175 residue of KDR in SHP-1 siRNA treated HUVEC compared to the scrambled control (Fig 5B), but only in the 5 min treatment window (Image quantitation data, see Additional file 3). Collectively, these data suggest that SHP-1 modulates some specific tyrosine residues of KDR that ultimately influences VEGF mediated DNA synthesis, but not migration.

### Modulation of KDR Phosphorylation and DNA Synthesis by c-Src

**Figure 7:**
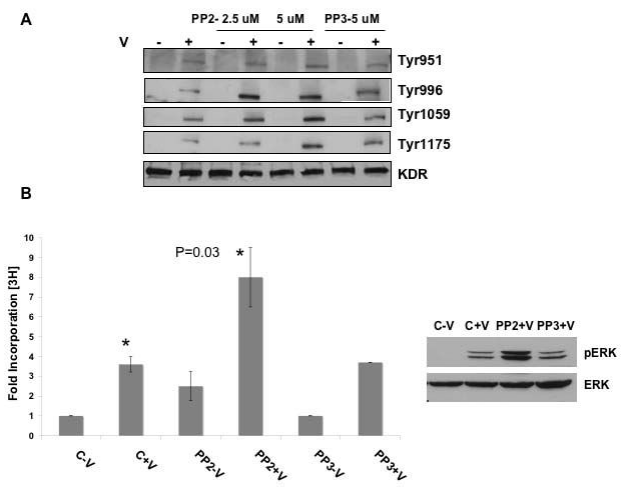
**Effect of inhibition of c-Src on phospho-Tyr of KDR and DNA synthesis**. (A) HUVEC was pretreated with PP2 or PP3 at 2.5 or 5 uM for 1 h and then with or without VEGF 10 ng/ml and immunoblotted with antibodies against p-Tyr of KDR. Total KDR served as loading control. (B) HUVEC were plated in 24 well plates, pretreated with PP2 or PP3 at 5 uM for 1 h and then with or without VEGF 10 ng/ml and [^3^H]thymidine incorporation carried out as described in Materials and Methods. Another set of the same treatment was subjected to a western blot for pERK and total ERK. Data represent average of three independent determinations each in triplicate (P = 0.03).

**Figure 8:**
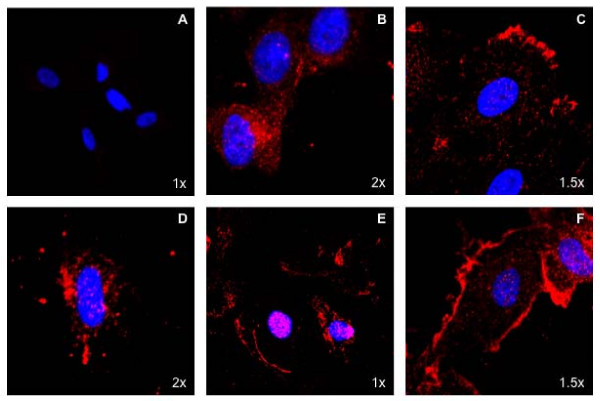
**Localization of p-Tyr 996 of KDR**. (A) HUVEC was treated with VEGF 10 ng/ml for 5 min and then stained with boiled p-Tyr 996 KDR antibody, this served as a negative control. (B) Untreated HUVEC was stained with p-Tyr 996 KDR antibody in red. (C) HUVEC treated with VEGF 10 ng/ml for 5 min was stained with p-Tyr 996 KDR antibody in red. (D) HUVEC was treated with bFGF 20 ng/ml and then stained with p-Tyr 996 KDR antibody in red. (E) HUVEC was pretreated with PP2 at 5 mM for 1 h and then stained with p-Tyr 996 KDR antibody in red. (F) HUVEC was pretreated with PP2 at 5 mM for 1 h and then with VEGF 10 ng/ml for 5 min and stained with p-Tyr 996 KDR antibody in red. The nuclei of the cells are stained with DAPI and appear blue. Magnification of images is noted in the lower right corner.

We next examined whether inhibition of c-Src kinase in VEGF-induced EC also mimics tyrosine phosphoryation of specific KDR residues as well as increased DNA synthesis. Accordingly, HUVEC were treated with c-Src kinase inhibitor PP2 and its analog PP3 at a dose of 5 uM in the presence or absence of VEGF 10 ng/ml. Figure 7A clearly shows that phosphorylation of Tyr 996, 1059 and 1175 residues of KDR increased upon treatment with PP2 in the presence of VEGF whereas PP3 was unable to do the same (Image quantitation data, see Additional file 5). We also observed a significant increase in ERK phosphorylation and proliferation of HUVEC both in the presence and absence of VEGF as compared to that of controls upon treatment with PP2 at a dose of 5 uM but not when treated with PP3 (Fig 7B and see Additional file 6). Confocal microscopy experiments also revealed an increase in phospho Tyr996 staining of KDR upon treatment with VEGF and a further increase in membrane staining upon PP2 treatment (Fig 8C, 8F). This pattern was not observed with bFGF treatment (Fig 8D) or in cells without VEGF (Fig 8B) and specificity of the antibody was determined by staining with the boiled antibody (Fig 8A). Overall, the c-Src kinase inhibition data clearly corroborate the results obtained with SHP-1 siRNA transfection and support our hypothesis that c-Src, upon activation by VEGF activates SHP-1 phosphatase activity and therefore regulates KDR function.

### Modulation of KDR Phosphorylation by SHP-1 Tyrosine Mutants

**Figure 9:**
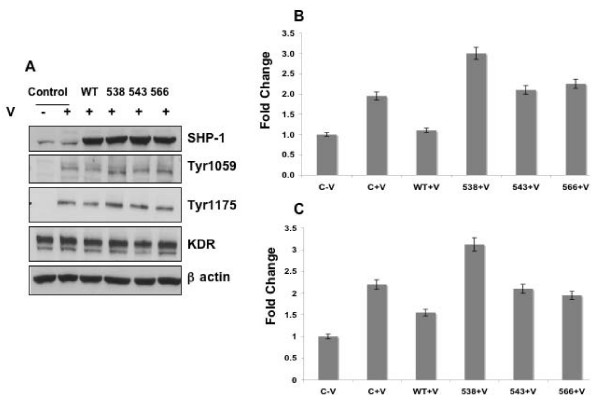
**Effect of SHP-1 mutations on de-phosphorylation of tyrosine residues on KDR**. (A) HUVEC was transfected with the following plasmids control pCDNA (C) or SHP-1 wildtype (WT) or SHP-1 Y538 or SHP-1 Y543 or SHP-1 Y566 mutants using nucleofector based electroporation. After 48 hrs. the cells were starved overnight in EBM medium without serum. Subsequently the cells were stimulated with VEGF for 5 min. Cell lysates were collected and expression of SHP-1 was confirmed. The same lysates were immunoblotted with antibodies against p-Tyr 1059 and p-Tyr 1175 of KDR. Total KDr and β-actin levels were also determined. NIH Image quantitation data of (B) fold change in Tyr 1059 and (C) fold change in Tyr 1175 of KDR normalized with respect to total KDR. A representative image is shown the experiment was repeated three times.

To determine the target tyrosine residues on KDR that could be dephosphorylated by SHP-1, we next overexpressed wild-type (WT) or the respective tyrosine mutants of SHP-1, namely, Y538, Y543 and Y566 in HUVEC. Overexpression was confirmed by western blot (Fig 9A). We observed that compared to the control cells stimulated with VEGF, phosphorylation of both Tyr1059 and Tyr1175 on KDR decreased in cells expressing the WT protein and increased significantly in cells expressing Y538 mutant SHP-1 protein (Fig 9B,9C). The other SHP-1 mutants such as Y543 or Y566 did not significantly modulate tyrosine phosphorylation of KDR. Therefore we infer that Y538 on SHP-1 is essential for dephosphorylation of tyrosine residues on KDR following VEGF stimulation.

## Discussion

VEGF is a critical regulator of angiogenesis, primarily through the signaling mediated by KDR. Although the signaling pathways *via *KDR have been extensively studied, the role of key proteins that modulate and 'fine-tune' VEGF signaling remains to be elucidated. In the present study, we have defined the specific tyrosine residues on KDR that are dephosphorylated by SHP-1 and modulate VEGF-mediated signaling. We also show that this dephosphorylation of tyrosine residues on KDR occurs upon stimulation with VEGF resulting in the activation of c-Src kinase and tyrosine phosphorylation of SHP-1. Our results are supported by the fact that the inhibition of c-Src kinase resulted in increased immunostaining of phospho-KDR with a concomitant increase in HUVEC proliferation and pERK. Our data clarifies a previously unexplained increase in phospho-KDR levels upon treatment with PP2 as described by Labrecque et al [35].

SHP-1 and KDR and KDR and c-Src co-immunoprecipitated with each other and the association between SHP-1 and KDR remained unchanged with or without VEGF treatment. Prior studies have indicated that VEGF induces association of SHP-1 with KDR. However our data consistently suggested that SHP-1 associates with KDR regardless of VEGF treatment. We presumed that in the culture conditions even after overnight starvation KDR remains phosphorylated at low levels leading to association with SHP-1. More importantly our data suggest that it is perhaps not the association but the activation of the tyrosine phosphatase activity of SHP-1 that is critical in regulating KDR-mediated downstream signaling. Nonetheless, the association of KDR with c-Src increased upon treatment with VEGF in HUVEC. Our results are in complete accordance with prior studies that have reported preferential association of c-Src with KDR upon stimulation with VEGF [36].

Knockdown of SHP-1 by siRNA in HUVEC caused a significant and consistent increase in proliferation after treatment with VEGF. These results suggest that the phosphatase activity of SHP-1 potentiates upon VEGF treatment. Previous reports suggested that the activity of SHP-1 is regulated by phosphorylation of the tyrosine and serine residues in the C terminus of the protein [20]. SHP-1 is phosphorylated on serine residues, constitutively in resting murine T cells. In human platelets it is phosphorylated on both tyrosine and serine residues in response to thrombin [37]. This is PKC dependent and correlates with an increase in the activity of SHP-1. In platelets, PKCα is constitutively bound to SHP-1 and PKCα dependent serine phosphorylation at 591 led to a decrease in phosphatase activity. However our results suggest that serine phosphorylation of SHP-1 decreases upon VEGF stimulation. SHP-1 is also phosphorylated on Tyr536 by the insulin receptor tyrosine kinase and this induces activation of its phosphatase activity [38]. A recent report by Frank et al., showed that c-Src is able to tyrosine phosphorylate SHP-1 *in vitro*, leading to an increase in the activity of the phosphatase [39]. Phosphorylation of SHP-1 at tyrosine 538 was required for optimal phosphatase activity of SHP-1 [39]. Therefore our observation of increased KDR phosphorylation upon mutation of Y538 of SHP-1 compared to the control in VEGF stimulated cells is substantiated by the previous reports. We also observed an increased c-Src dependent tyrosine phosphorylation of SHP-1 and inhibition of c-Src kinase activity by PP2 caused a significant increase in DNA synthesis as well as phospho-KDR (Y996) staining on HUVEC. It should be mentioned here that the relative increase in DNA synthesis after inhibiting c-Src kinase compared to knockdown of SHP-1 was much more potent. This is probably because c-Src negatively regulates a number of other proteins including caveolin-1 and dynamin that may play a role in proliferation [35,40]. We also identified specific tyrosine residues on KDR that were phosphorylated more in SHP-1 knockdown and PP2 treated cells. Migration, as determined by the wound healing assay or phospho-KDR (Y951) a marker for migration, was not significantly affected while proliferation markers like phospho-Y996, Y1059 and Y1175 were significantly more phosphorylated. An increase in phospho-ERK was also observed in SHP-1 siRNA treated cells. This finding is supported by a previous study that showed activation of ERK1/2 after using a dominant-negative inhibitor of SHP-1 [41].

It has been previously shown that in 293T cells Tyr996 of KDR perhaps remains phosphorylated as no difference is observed in the phosphopeptide map after addition of VEGF (12). However, in our experiments we repeatedly find Tyr996 of KDR to be phosphorylated in a VEGF dependant manner. This has been confirmed by Western blotting as well as confocal microscopy. We also confirmed specificity of the antibody by staining with or without bFGF treatment. We found clear, distinct membrane staining after treatment with VEGF but not bFGF. This leads us to speculate that HUVEC being endothelial cells differ from 293T in the way they respond to VEGF or that Tyr996 might not be autophosphorylated and could be phosphorylated by some other kinase upon activation with VEGF.

However, the question remains why SHP-1 with the help of c-Src kinase controls KDR-mediated proliferation in HUVEC. In order for proper vascular remodeling to occur, fine-tuning of proliferative and/or anti-proliferative cues controlling KDR-induced downstream signaling is expected to be required and with VEGF as a potential endothelial mitogen, it is a logical candidate. In this regard, other than its role in vascular hyperpermeability [42], VEGF-mediated c-Src kinase activation, leads to a better organization of vascular structure by controlling KDR function. Future studies are in progress to resolve the current hypothesis. Nonetheless, our study illustrates a new role for c-Src as well as SHP-1 in VEGF-induced angiogenesis.

## Conclusion

The main conclusions are: 1. KDR, SHP-1 and c-Src are part of the same immnocomplex. 2. The phosphatase activity of SHP-1 increases after VEGF treatment and is Src dependent. 3. Inhibition of Src in endothelial cells causes increased tyrosine phosphorylation of VEGFR-2 and pERK ultimately leading to increased proliferation. 4. We identified the specific tyrosine residues on KDR that are modulated by SHP-1. 5. We identified Y538 on SHP-1 to be the major site that regulates its phosphatase activity and hence is responsible for de-phosphorylation of tyrosine residues on VEGFR-2 subsequent to VEGF stimulation.

## Methods

### Reagents

VEGF-A was obtained from R&D systems, Minneapolis, MN. [^3^H] Thymidine was from Amersham Biosciences. The antibodies to KDR, c-Src, phosphoKDR (996) and phosphoKDR (951) were from Santa Cruz Biotechnology (Santa Cruz, CA); phosphoKDR (1059) was from Upstate and phospho KDR (1175) from Cell Signaling. PP2 and PP3 were from EMD Biosciences (San Diego, CA). The antibodies to SHP-1 were from Santa Cruz Biotechnology (Santa Cruz, CA) and BD-Biosciences (San Jose, CA).

### Immunoflourescence

Anti-KDR monoclonal antibody used for immunoflourescence was from Sigma-Aldrich, St. Louis, MS. AlexaFlour 488 anti-mouse or AlexaFlour 546 anti-rabbit secondary antibody was from Molecular probes, Eugene, OR. 2 × 10^4 ^HUVEC were seeded on collagen coated Lab-Tek chamber slides. After 24 h cells were serum starved. The next day cells were treated with or without VEGF 10 ng/ml. Slides were washed in PBS, fixed in 4% paraformaldehyde (PFA) and permeabilized with 0.2% TRITON X-100 at room temperature. Slides were washed in PBS, blocked in 10% goat serum and stained with respective primary antibodies in 1% goat serum for 2 hrs. Slides were washed in PBS and incubated for 1 h in respective secondary antibody at a dilution of 1:200 followed by post-fixing in 4% PFA and mounting in Vectashield, Vector Labs, CA. Confocal microscopy was performed using a Zeiss LSM 510 confocal laser scan microscope with C-Apochromat 63×/NA 1.2 water-immersion lens. Absence of signal crossover was established using single-labeled samples.

### Immunoprecipitation and Western Blot Analysis

Serum starved HUVEC were treated with or without VEGF 10 ng/ml and/or PP2 or PP3 5 uM. Cell lysates in RIPA buffer supplemented with protease inhibitor cocktail were prepared from HUVEC. The lysates were collected after centrifugation at 14000 × g for 10 min at 4°C. 500 ug of lysate protein was incubated with 1 mg respective antibody for 1 h and 50 ml of proteinA/G-conjugated agarose-beads for an additional hour at 4°C. Beads were washed with RIPA buffer three times and immunoprecipitates were resuspended in 2× SDS sample buffer.

HUVEC were transfected with the following plasmids control pCDNA (C) or SHP-1 wildtype (WT) or SHP-1 Y538 or SHP-1 Y543 or SHP-1 Y566 mutants using nucleofector based electroporation. The above plasmids were a kind gift from Dr. Z. Yu (National Research Council, Canada). After 48 hrs. the cells were starved overnight in EBM medium without serum. Subsequently the cells were stimulated with VEGF 10 ng/ml for 5 min. Cell lysates were collected and immunoblotted with respective antibodies. Please note that immunoprecipitation or western blot as shown in Fig. 1 through Fig. 5 was performed exactly as described here but without any overexpression of SHP-1, KDR or Src rather on endogenous proteins from HUVEC lysates. Experiments were repeated at least three times.

### Cell Proliferation Assay

HUVECs (2 × 10^4^) were seeded in 24-well plates and cultured for 24 h in EGM. After 24 h the cells were serum starved and pre-treated with or without PP2 or PP3 at 5 mM for 1 h respectively before stimulation with VEGF 10 ng/ml. After culture for 20 h, 1 mCi of [^3^H]thymidine was added to each well; 4 hrs later, cells were washed with chilled PBS, fixed with 100% cold methanol and collected for measurement of trichloroacetic acid-precipitable radioactivity. Experiments were repeated at least three times each time in triplicate.

### siRNA Transfection

1 × 10^5 ^HUVEC were seeded in 60 mm plates and cultured for 24 h in EGM. The next day cells were washed with OPTI-MEM reduced serum medium and transfected with 50 nM SHP-1 siRNA obtained from Santa Cruz Biotechnology (sc29478) using oligofectamine (Invitrogen). This siRNA is a pool of 3 sequences. Sense Strand (A):CUGGUGGAGCAUUUCAAGATT, (B):CGCAGUACAAGUUCAUCUATT and (C): CAACCCUUCUCCUCUUGUATT. After 4 hrs antibiotic free EGM was added and cell lysates were prepared 48 hrs after transfection.

### SHP-1 Phosphatase Assay

Phosphatase assay was performed according to manufacturers protocol (Stratagene, Signal Scout Phasphatase Profiling System). Briefly, HUVEC were pretreated for 1 h with 5 mm PP2 or PP3 and then with or without 10 ng/ml VEGF for 5 min. Cells were lysed (100 mM NaCl, 10 mM Tris-HCl pH 8.0, 1 mM EDTA, 1% Triton ×, 5 mM DTT), lysate pre-cleared and protein quantity determined by Bradford assay. Equal amount of protein from each sample was then immunoprecipitated with a SHP-1 antibody using the "catch and release column" from Upstate. One part was then subjected to western blot and another part was treated as follows; 80 ul complete assay buffer (14 mM HEPES pH 7.4, 30 mM NaCl, 1.5 mM EDTA, 5 mM DTT) was added and eluted from the column. 120 ul pNPP substrate (20 mM) was then added to each sample and absorbance at 405 nm was read after 30 min incubation at 30°C on a TECAN Spectra Flour Plus. Data presented were normalized with respect to negative control. Three independent experiments were performed.

### Wound Healing Migration

Monolayers of HUVEC transfected with scrambled control or SHP-1 siRNA were scratched with a universal blue pipette tip and incubated for 12 hrs in the presence of 10 ng/ml VEGF. Thymidine (10 mM; Sigma-Aldrich) was included during the incubation to inhibit cell proliferation. Migration of cells across the scratched area was recorded by time-lapse microscopy (Apotome, Carl Zeiss) using AXIO Vision software. Cells were counted from five fields per well.

### Statistical Analysis

All values are expressed as means ± SD. Statistical significance was determined using two-sided Student's t test, and a value of P < 0.05 was considered significant. For Figure 5B, ANOVA was calculated using Tukey's Studentized Range (HSD) Test. Comparisons significant at the 0.05 level are shown (Supplemental data).

## Abbreviations

Abbreviations used are: SHP-1, Src homology 2 domain containing protein tyrosine phosphatase-1; SHP-2, Src homology 2 domain containing protein tyrosine phosphatase-2; VEGF, Vascular endothelial growth factor; KDR, Vascular endothelial growth factor receptor-2; siRNA, silencing RNA; Human umbilical vein endothelial cells, HUVEC; Mitogen activated protein kinase, ERK.

## Competing interests

The author(s) declare that they have no competing interests.

## Authors' contributions

RB conceived the project and performed majority of the experiments with significant help from others as stated. JK participated in the siRNA transfections and wound healing migration assays. EW performed most of the western blots. PM participated in the phosphatase assay and proliferation assay. DM participated in the conception of the study, design and coordination and helped to draft the manuscript.

## Supplementary Material

Additional file 1 Determining specificity of SHP-1 antibody. (A) SHP-1 was immunoprecipitated from HUVEC lysates using an antibody and the "Catch and release column" from Upstate. It was immunostained with an HRP-conjugated secondary antibody to SHP-1. (B) SHP-1 was immunoprecipitated as above. It was immunostained with an antibody to SHP-2. Click here for file

Additional file 2 NIH Image quantitation data. Supplement of Fig 5A. Fold change in Tyr 951 (A), Tyr 996 (B), Tyr 1059 (C), and Tyr 1175 (D) of KDR normalized with respect to total KDR. Click here for file

Additional file 3 NIH Image quantitation data. Supplement of Fig 5B. (A) Fold change in pERK normalized with respect to total ERK. (B) Fold change in Tyr 1175 of KDR normalized with respect to total KDR. Click here for file

Additional file 4 Quantitation of scratch migration assay in HUVEC. Supplement of Fig 6. Average number of cells migrated into the wounded area after 12 h. Click here for file

Additional file 5 NIH Image quantitation data. Supplement of Fig 7A. Fold change in Tyr 951 (A), Tyr 996 (B), Tyr 1059 (C) and Tyr 1175 (D) of KDR normalized with respect to total KDR. Click here for file

Additional file 6 Detailed statistical analysis of data presented in Fig 7B. Shows ANOVA, Tukey's Studentized Range Test (HSD) comparisons between different groups tested. Click here for file
