# Supplementary figures and images for: Src homology 2 (SH2) domain containing protein tyrosine phosphatase-1 (SHP-1) dephosphorylates VEGF Receptor-2 and attenuates endothelial DNA synthesis, but not migration*
Source: J Mol Signal. 2008 Mar 31;3:8. doi: 10.1186/1750-2187-3-8 (PMC2292718; doi:10.1186/1750-2187-3-8)

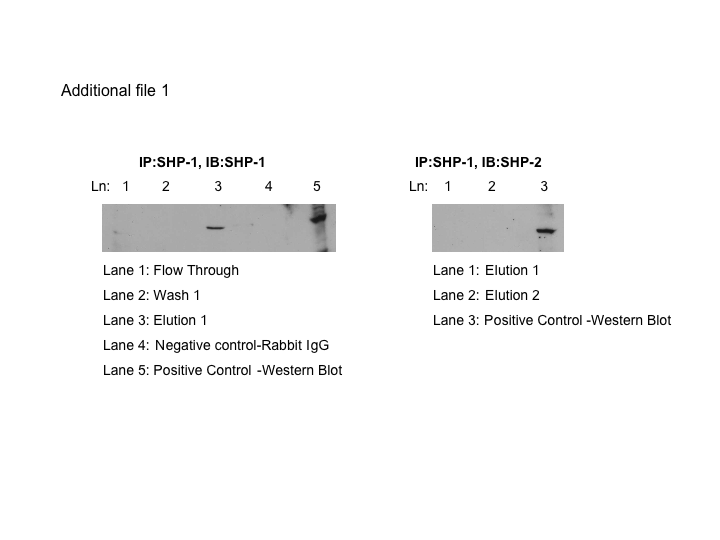

Supplement: Additional file 1 — Determining specificity of SHP-1 antibody. (A) SHP-1 was immunoprecipitated from HUVEC lysates using an antibody and the "Catch and release column" from Upstate. It was immunostained with an HRP-conjugated secondary antibody to SHP-1. (B) SHP-1 was immunoprecipitated as above. It was immunostained with an antibody to SHP-2. [file 1750-2187-3-8-S1.tiff]

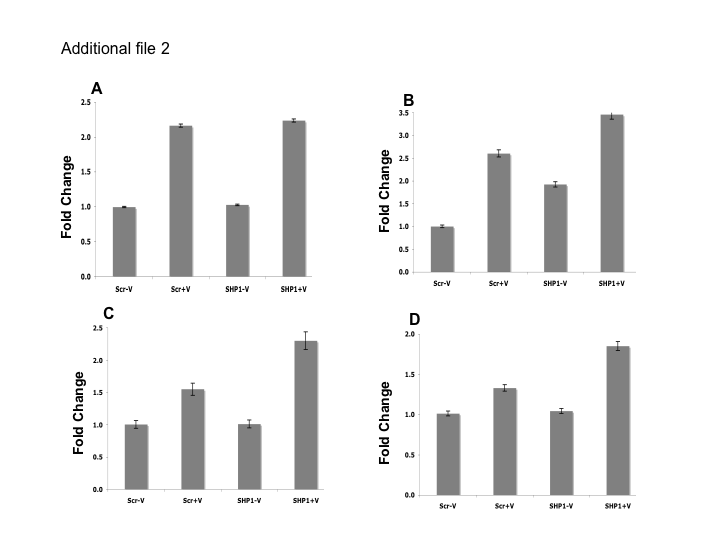

Supplement: Additional file 2 — NIH Image quantitation data. Supplement of Fig 5A. Fold change in Tyr 951 (A), Tyr 996 (B), Tyr 1059 (C), and Tyr 1175 (D) of KDR normalized with respect to total KDR. [file 1750-2187-3-8-S2.tiff]

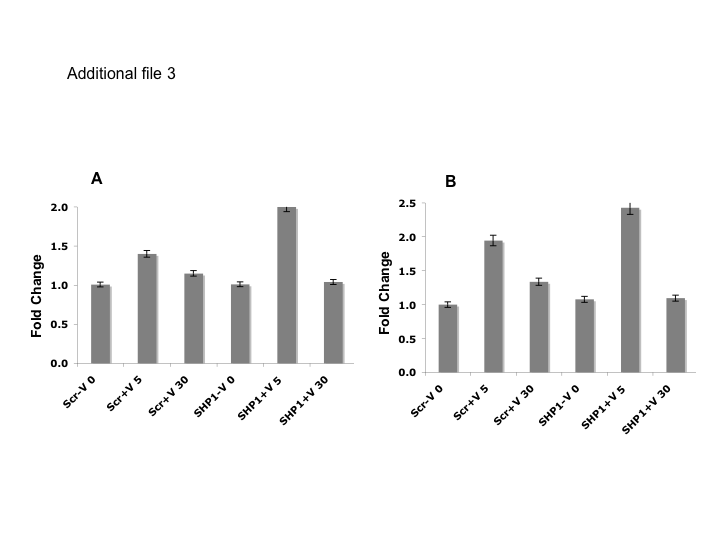

Supplement: Additional file 3 — NIH Image quantitation data. Supplement of Fig 5B. (A) Fold change in pERK normalized with respect to total ERK. (B) Fold change in Tyr 1175 of KDR normalized with respect to total KDR. [file 1750-2187-3-8-S3.tiff]

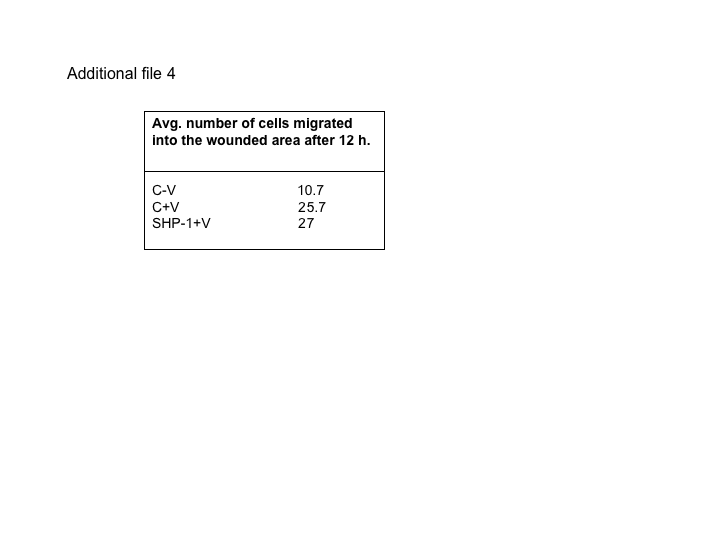

Supplement: Additional file 4 — Quantitation of scratch migration assay in HUVEC. Supplement of Fig 6. Average number of cells migrated into the wounded area after 12 h. [file 1750-2187-3-8-S4.tiff]

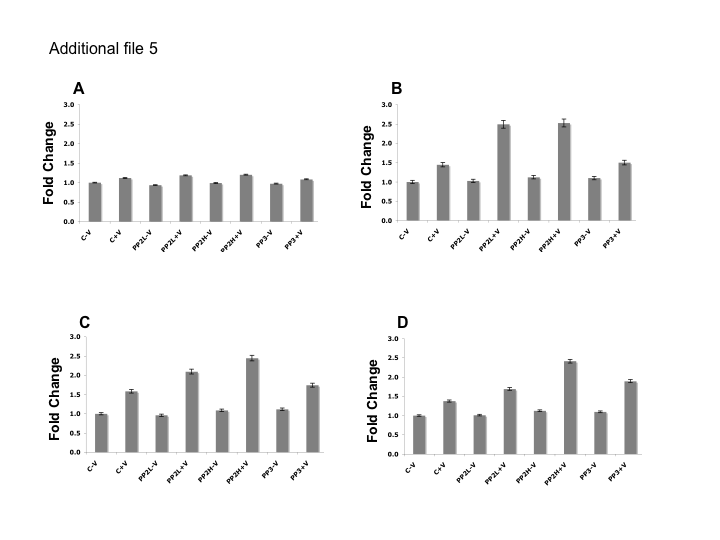

Supplement: Additional file 5 — NIH Image quantitation data. Supplement of Fig 7A. Fold change in Tyr 951 (A), Tyr 996 (B), Tyr 1059 (C) and Tyr 1175 (D) of KDR normalized with respect to total KDR. [file 1750-2187-3-8-S5.tiff]

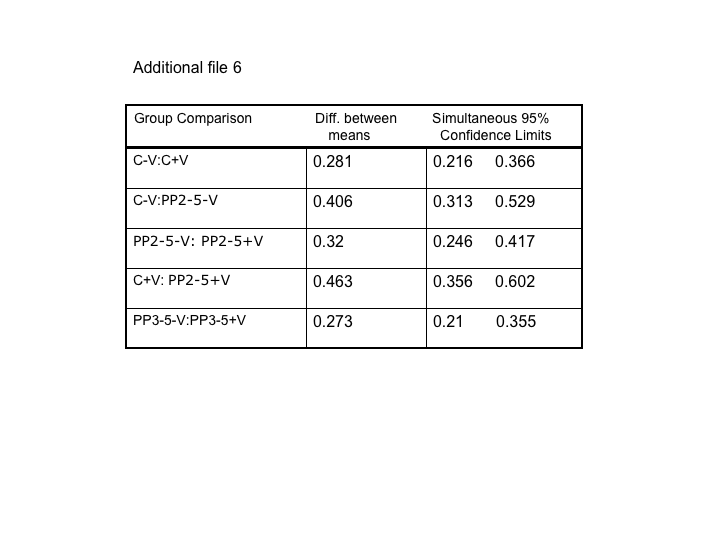

Supplement: Additional file 6 — Detailed statistical analysis of data presented in Fig 7B. Shows ANOVA, Tukey's Studentized Range Test (HSD) comparisons between different groups tested. [file 1750-2187-3-8-S6.tiff]
